# Infantile Aggressive Aneurysmal Bone Cyst of the Proximal Femur: A Rare Clinical Presentation

**DOI:** 10.7759/cureus.75708

**Published:** 2024-12-14

**Authors:** Furkan Erdoğan, Tolgahan Cengiz, Şafak Aydın Şimşek, Cahit Şemsi Şay, Hüseyin Sina Coşkun, Nevzat Dabak

**Affiliations:** 1 Orthopaedics and Traumatology, Faculty of Medicine, Ondokuz Mayıs University, Samsun, TUR

**Keywords:** aneurysmal bone cyst, bone curettage, osteolytic lesion, pathological fracture, proximal femur

## Abstract

Aneurysmal bone cysts (ABCs) are aggressive, osteolytic lesions usually seen in childhood and young adulthood. The patient's age, location, and behavior of the lesion in the bone may cause patients to present with different clinical findings. Appropriate treatment of these rare, aggressive bone lesions is essential for recurrence. This case report aims to present the diagnosis and treatment approach in the case of an infantile aggressive ABC and to present the long-term follow-up results.

A 14-month-old baby boy was admitted to the clinic with the complaint of difficulty in walking after a fall. After evaluation with advanced imaging methods, a fluid-filled cystic lesion and pathological fracture were detected in the proximal femur. After surgical treatment of the lesion, which showed an aggressive course in the follow-up, the patient was able to give a whole load six months after surgery. Although the ABC was aggressive in this case, it was controlled with surgical treatment and long-term follow-up. It should be kept in mind that ABCs in children, especially in the proximal femur, may be aggressive and may require surgical treatment.

## Introduction

Aneurysmal bone cysts (ABCs) are benign but aggressive bone lesions that usually occur in the first two decades of life. They are most commonly found in the metaphysis of long bones and the spine. ABCs are responsible for around 1% of all bone cancers, with reported incidences of 0.14 and 0.32 instances per 100,000 population, respectively [1]. ABCs generally appear in childhood and young adulthood, with a median age of 13 years, 90% of lesions detected before 30 years, and a male-to-female sex ratio of 1:1.16 [2].

ABCs may typically present with pain, swelling, or a palpable mass. Differences in the growth rate and location of ABCs can lead to different clinical presentations. Acute pain may occur if a pathological fracture develops [3]. Neurological symptoms can also occur if the skeleton is in close proximity to nerves [4].

There are several theories regarding the etiology of ABCs. Although they are expected to form primary tumors, they are also believed to develop as lesions secondary to trauma or secondary to main bone tumors such as giant cell tumors or chondroblastoma [5].

It is an enlarged, radiolucent lesion often situated in the metaphyseal part of the bone. Magnetic resonance imaging shows fluid-filled levels. When an ABC is suspected, it is difficult to distinguish it from telangiectatic osteosarcoma, which can seem similar to ABCs histologically and radiographically, as well as in age and clinical presentation. Treatment usually consists of curettage and grafting for benign bone tumors, but adjuvant therapies are often used to prevent a possible recurrence.

In this case report, we aimed to share the diagnostic process and treatment algorithm of an ABC in the proximal femur of a 14-month-old boy.

## Case presentation

A 14-month-old male infant was admitted to the clinic with complaints of difficulty walking and stepping on the right lower extremity due to a fall 1.5 months earlier. On physical examination, bilateral lower extremity neurovascular examination was normal. The patient's right hip movements were limited to active-passive, and passive joint movement examination revealed avoidance and pain response. The patient had no known comorbidity but had a history of a fall from the same level 1.5 months ago.

**Figure 1:**
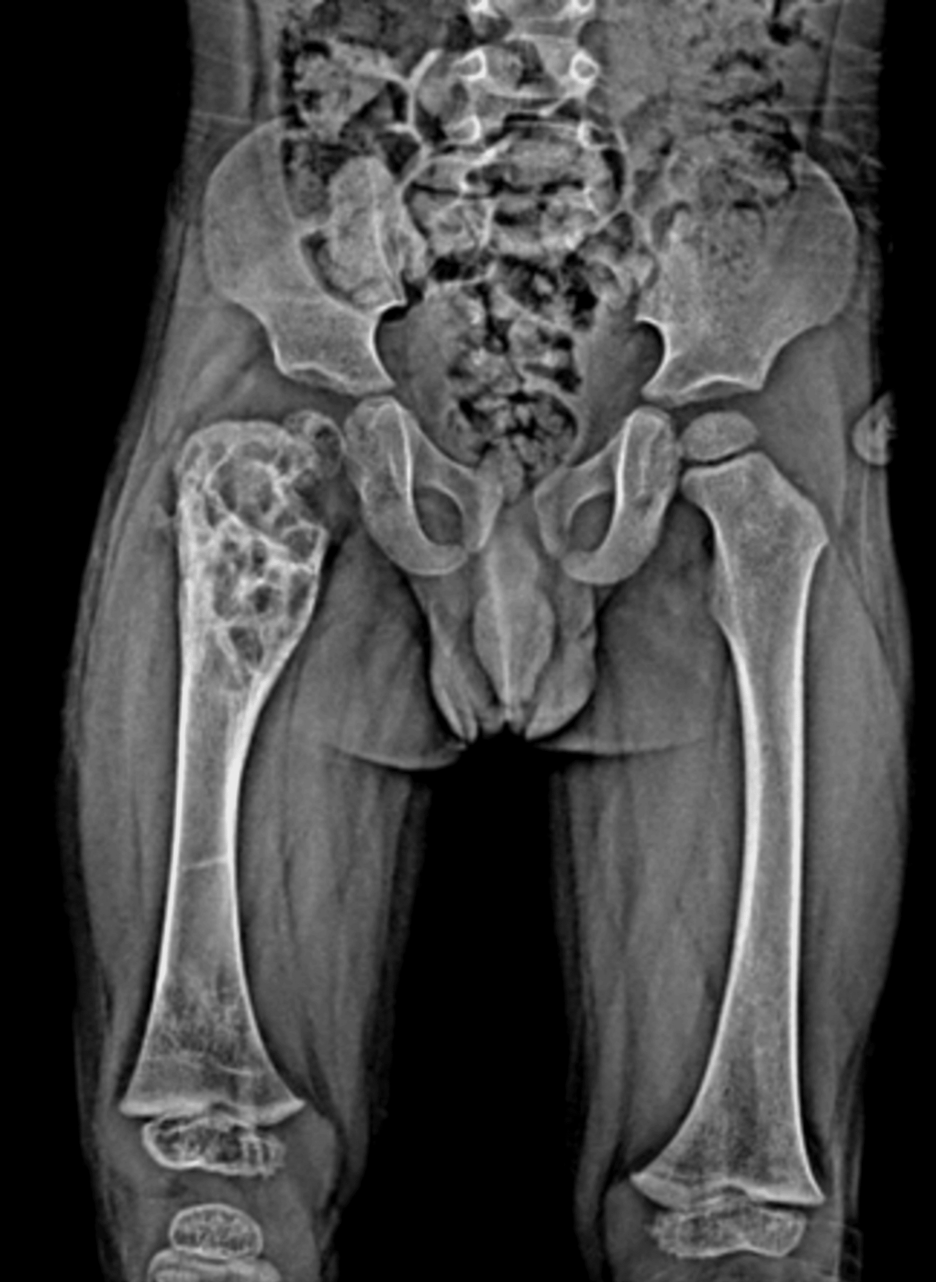
Pathologic fracture in the proximal metaphysis of the right femur on the anteroposterior radiograph of the pelvis

**Figure 2:**
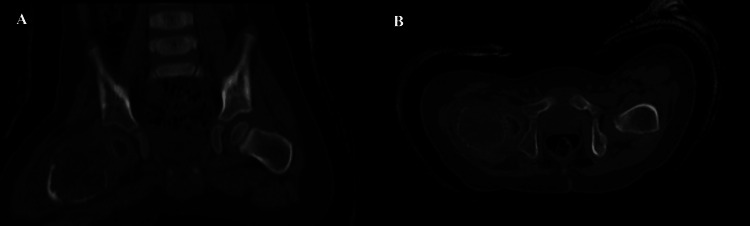
CT coronal (A) and axial (B) sections show a 49x36 mm hypodense cystic expansile lesion with a sclerotic margin in the proximal metaphysis of the right femur

**Figure 3:**
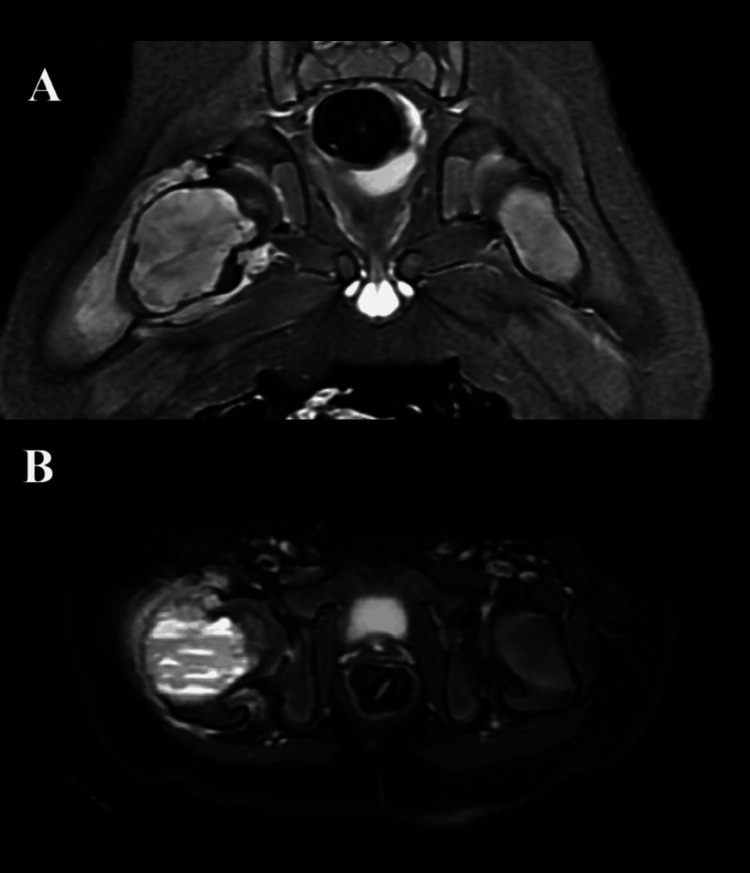
MR coronal (A) and axial (B) T2 sequence imaging showing a cystic mass lesion in the proximal metaphysis of the right femur with expansion and fluid-fluid leveling leading to pathological fracture

An anteroposterior pelvic radiograph (Figure 1) showed a cystic lesion with a suspected pathological fracture in the proximal metaphysis of the right femur, and further investigation was planned. Computed tomography (CT) (Figure 2) showed a 49x36 mm hypodense mass lesion with a sclerotic margin in the proximal metaphysis of the right femur, which was considered an expansile cystic lesion. Magnetic resonance imaging (MRI) (Figure 3) showed an expansile mass lesion of a cystic nature in the proximal metaphysis of the right femur with a pathological fracture with fluid-fluid leveling and reactional edema around it.

**Figure 4:**
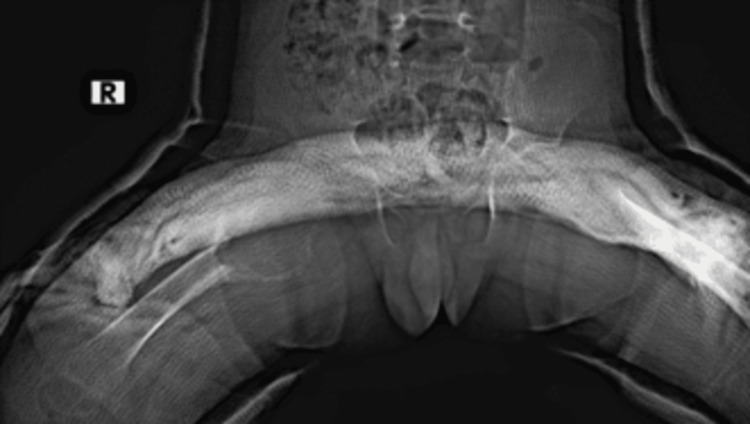
First-month control radiograph after pelvipedal cast

**Figure 5:**
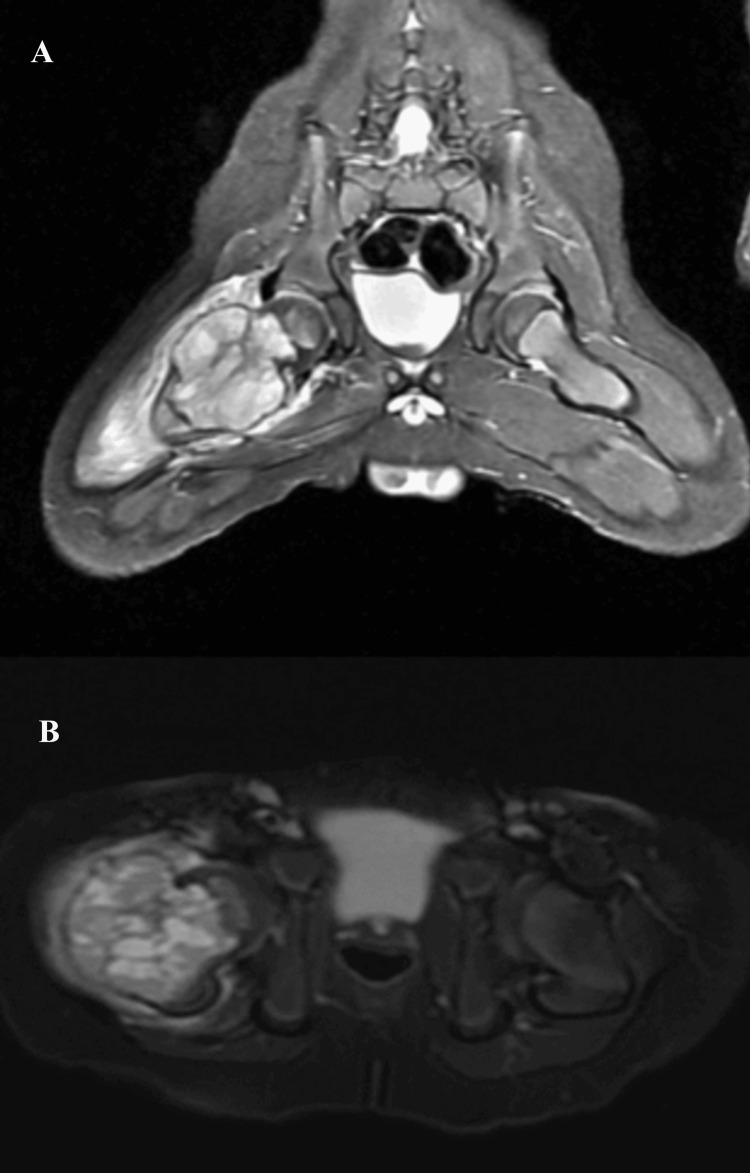
T2 hyperintense mass lesion with lobulated contour and septa formations increasing to 51x43 mm in size

**Figure 6:**
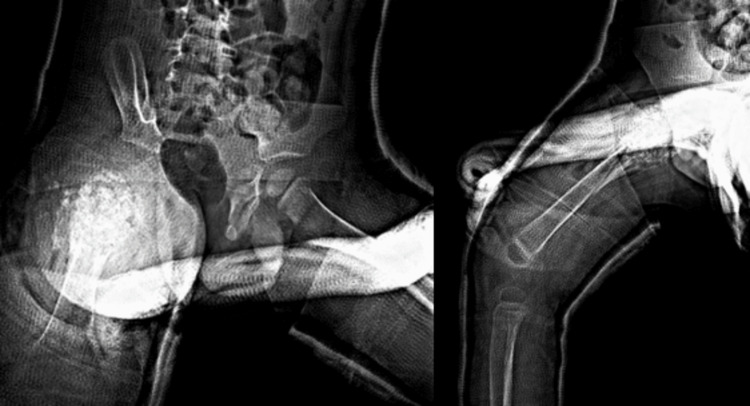
Anteroposterior and lateral radiographs intraoperatively

**Figure 7:**
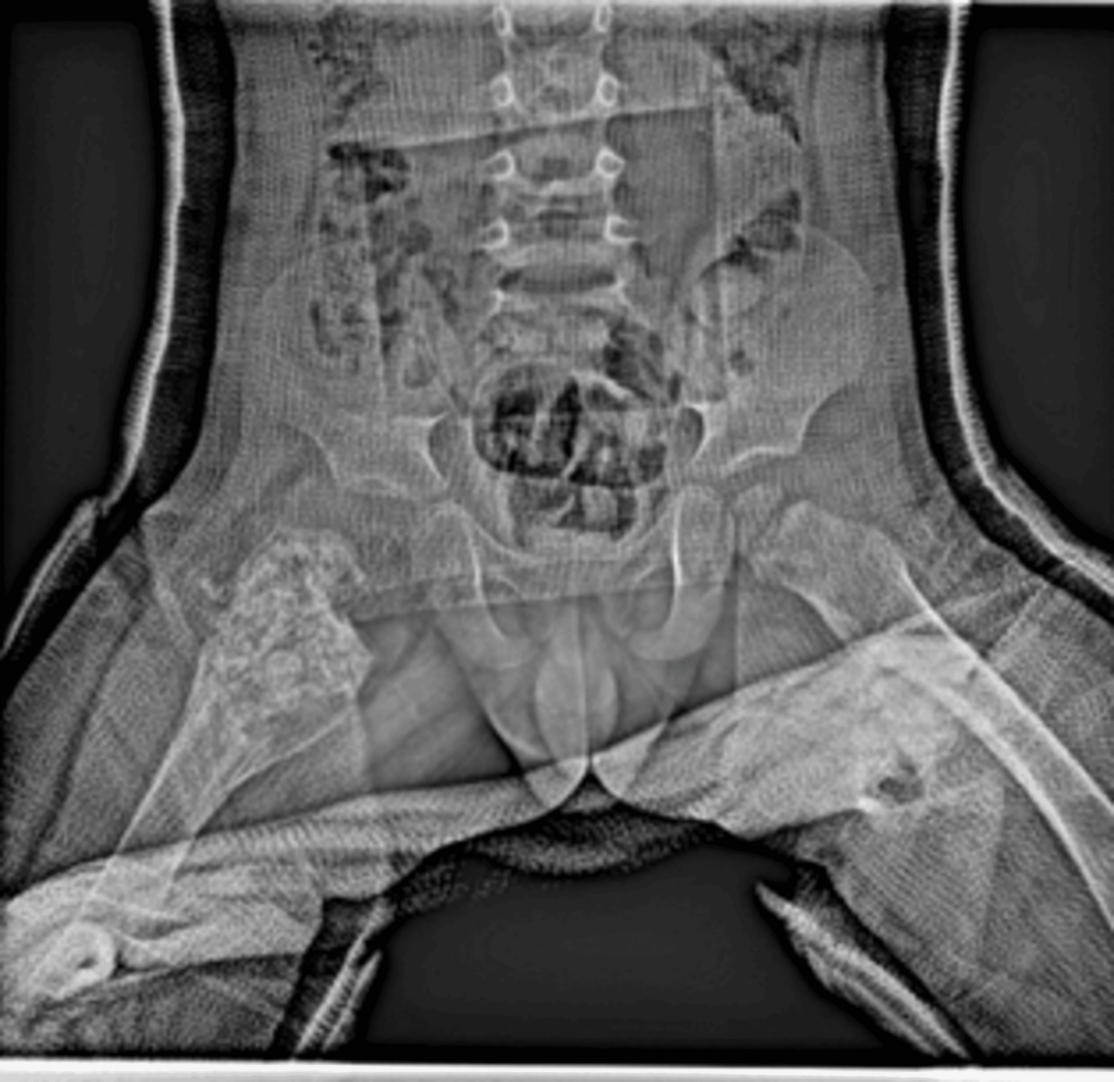
Postop sixth-week control radiograph

**Figure 8:**
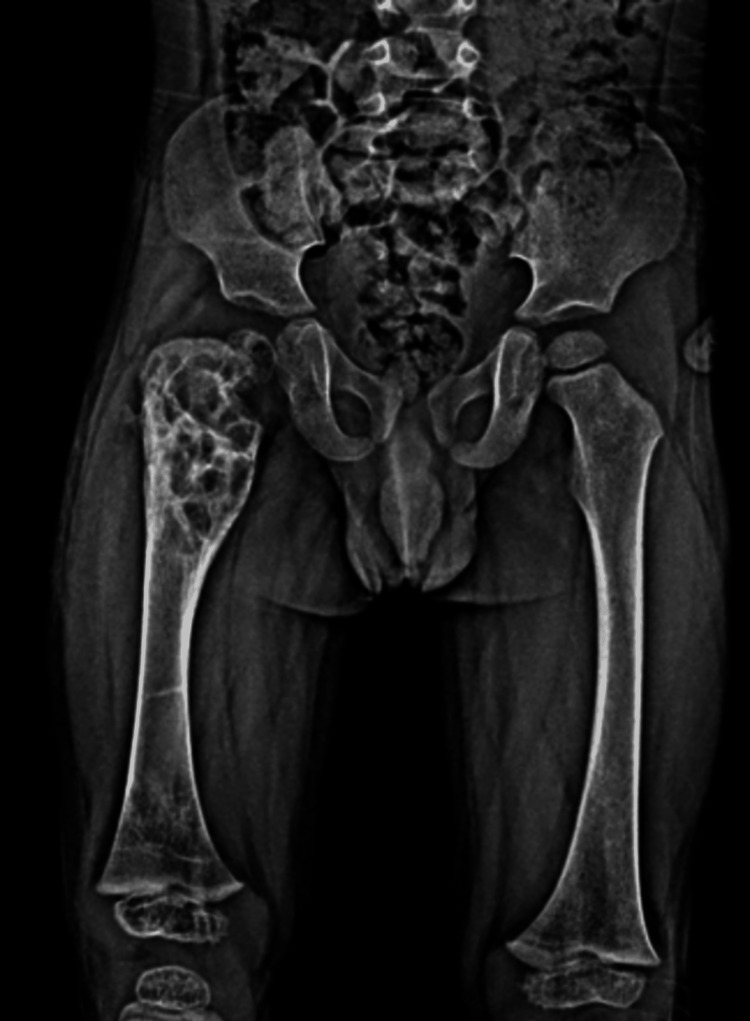
Sixth-month control radiograph of a clinically asymptomatic patient

After the patient was evaluated in the multidisciplinary tumor council, it was decided to apply a pelvipedal cast and close clinical and radiological follow-up. In the first month follow-up, it was decided to use surgical treatment for the patient due to the increase in the size of the previously detected lesion in the control radiographs (Figures 4, 5). Intraoperative evaluation showed that the cortex of the proximal femur was destructed, the metaphysis was wholly affected, and there were extensive hemorrhagic lesion areas up to the epiphyseal line. Intralesional curettage followed by grafting was performed, and the material from the surgical field was sent for pathologic examination. After bleeding control and control imaging with scope (Figure 6), a pelvipedal cast was applied. After the sixth week follow-up (Figure 7), the pelvipedal cast was discontinued, and the hip's active and passive joint movements were started. In the six-month follow-up (Figure 8), the patient could walk with full load on the lower extremity.

**Figure 9:**
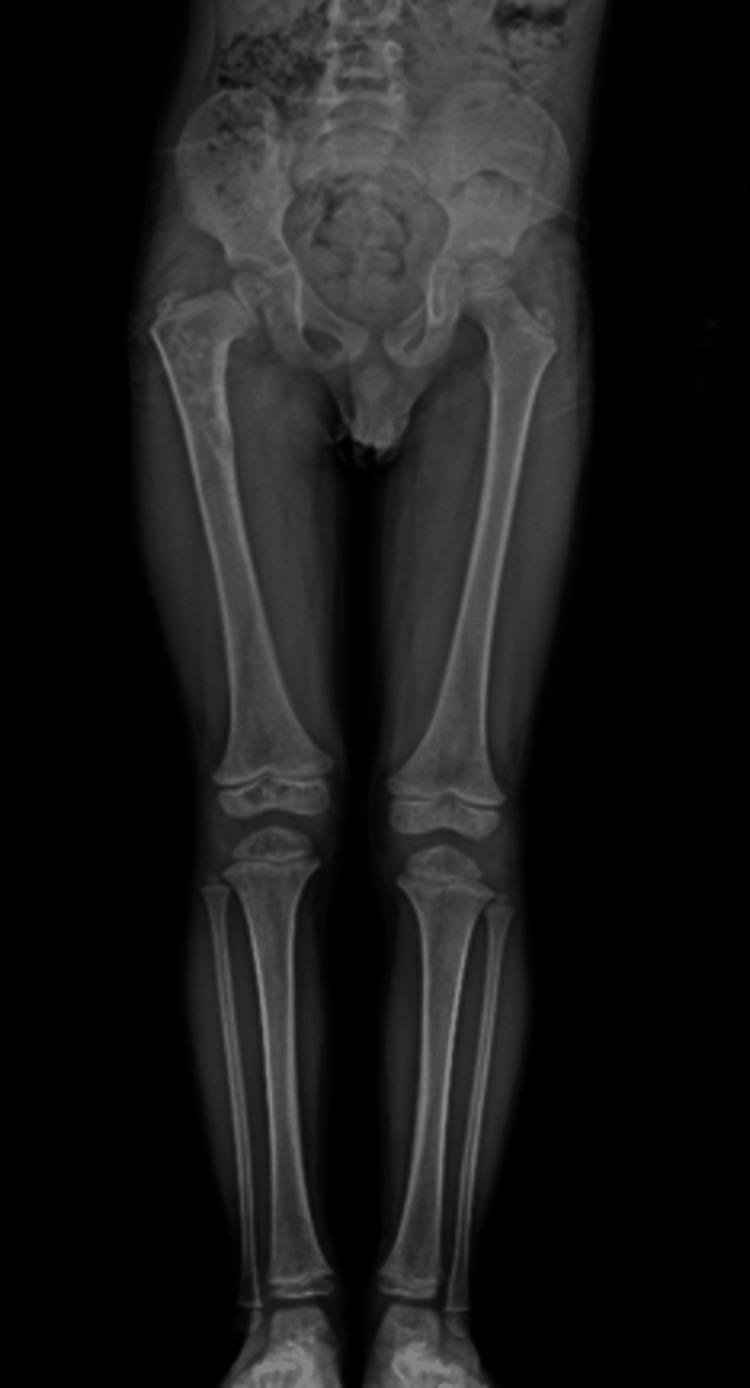
There is no evidence of an aneurysmal bone cyst on the third-year follow-up radiograph, but he is followed up because of coxa vara

The patient is now four years old, and there is no pathologic appearance of an ABC on the control radiograph (Figure 9). In addition, the patient has no active symptomatic complaints or leg length discrepancy but is under close clinical follow-up due to coxa vara.

## Discussion

ABCs are expansile and osteolytic lesions characterized by cystic cavities filled with blood elements and composed of fibroblasts, mononuclear cells, and osteoclastic giant cells. In more than half of the instances, they are located at the level of the long bones, primarily the femur, and tibia; in 12-30% of cases, the spine is damaged; and in the remaining cases, flat bones such as the pelvis are afflicted [3]. They can proliferate and weaken the bone, leading to pathological fractures. They are often asymptomatic but can cause acute symptoms of local swelling and pain. The differential diagnosis should consider simple bone cysts, chondromyxoid fibroma, giant cell tumors, chondroblastoma, osteoblastoma, and telangiectatic osteosarcoma. Caution should be exercised as it may be confused with telangiectatic osteosarcoma due to its age group, clinical presentation, and histological morphology [1].

In both ABCs and telangiectatic osteosarcoma, viable (atypical, stromal) cells are primarily found within the septa and on the perimeter of the cystic lesion, making it difficult to obtain an appropriate diagnostic sample during a biopsy. Fine needle aspiration biopsy may be an alternative because a solid specimen is frequently not obtained; however, its analysis takes considerable knowledge from a cytopathologist [6].

For definitive treatment, the tumor must be proven histologically. Because ABCs are lytic and aggressive lesions, they should be treated completely. Surgical treatment is frequently performed, and curettage is followed by grafting with or without adjuvant therapy [7]. Adjuvant treatment tries to treat microscopic contamination inside the tumor bed to lower the risk of local recurrence. To reduce intraoperative bleeding in big tumors affecting the axial skeleton and pelvis, preoperative embolization may be considered [8]. Factors associated with treatment success or failure have been examined in various studies, and young age at presentation, physis involvement, and previous treatment (recurrent cysts) have been identified as unfavorable prognostic indicators [9].

In the literature, ABCs have been reported in infantile cases, but their localization is usually in the spheno-orbital region. When they occur in the sphenoid bone, they may present with symptoms such as proptosis, headache, vision loss, and sometimes cranial nerve palsies [10].

The literature shows 5-50% recurrence rates in cases with active, aggressive course, central localization in long bones, and inadequate curettage after surgical treatment [11]. Furthermore, there were high recurrence rates for curettage alone (20%), while recurrence was reported to be significantly reduced when combined with cancellous bone graft (3%) [12]. The curettage technique is also of great importance in terms of recurrence. Recurrence rates as low as 10% have been reported after endoscopic or high-speed burr-assisted curettage procedures [13]. Various adjuvants such as phenol, hydrogen peroxide, defect reconstruction with polymethylmethacrylate, high-speed burr, or electrocautery are applied to minimize the risk of local recurrence [11]. Patients should be followed clinically and radiologically for a prolonged period to assess recurrence.

## Conclusions

In the present case, the ABC was quite aggressive in the planned follow-up surgical treatment, and the lesion was controlled in the long-term follow-up. In the literature, there are a limited number of cases of ABCs localized in the femoral region in children. It should be kept in mind that ABCs in the hip region, especially in the infantile age group, may have an aggressive course and need surgical treatment.
